# Prognostic differences in patients with advanced solid tumors receiving immune checkpoint inhibitors: The role of immune-related adverse events

**DOI:** 10.1097/MD.0000000000043153

**Published:** 2025-07-04

**Authors:** Cheng Zhao, Huiqian Liu, An Wang, Bin Zhai, Bin Jiang, Lina Hu, Hui Yu, Cui Bai, Kai Chen

**Affiliations:** a Department of Medical Oncology, Anqing Petrochemical Hospital of Nanjing Drum Tower Hospital Group, Anqing, Anhui Province, China.

**Keywords:** advanced solid tumors, adverse immune reactions, immune checkpoint inhibitors, treatment efficacy

## Abstract

This retrospective study examines the relationship between negative immune responses and the effectiveness of immune checkpoint inhibitors (ICIs) in the treatment of advanced solid tumors. A total of 59 patients with advanced solid tumors treated with ICIs between August 2020 and May 2022 were included. Based on the presence or absence of immune-related adverse events (irAEs), patients were categorized into 2 cohorts: irAE group (n = 46) and non-irAE group (n = 13). The primary objective was to compare therapeutic efficacy and survival outcomes between these cohorts. Among patients who developed immune-related adverse events (irAEs), the disease control rate (DCR) was 93.48% and the objective response rate (ORR) was 30.43%. In contrast, patients without irAEs exhibited significantly lower rates (DCR: 38.46%; ORR: 7.69%). Kaplan–Meier analysis demonstrated significantly prolonged progression-free survival (PFS) in the irAE group (median: 10.34 months; 95% CI: 9.275–11.401) compared to the non-irAE group (median: 5.82 months; 95% CI: 2.868–8.772; *P *< .05). Gastrointestinal malignancies (gastric cancer, HCC, and esophageal carcinoma) exhibited the most favorable PFS outcomes. Multivariate Cox regression analysis showed that irAEs, cutaneous irAEs, and endocrine irAEs were significantly associated with prolonged PFS. Multivariate Cox proportional hazards regression analysis identified irAE occurrence (hazard ratio [HR] = 0.542; 95% CI: 0.295–0.971; *P* = .04), cutaneous irAEs (HR = 0.476; 95% CI: 0.232–0.912; *P* = .03), and endocrine irAEs (HR = 0.237; 95% CI: 0.037–0.842; *P* = .02) as independent predictors of prolonged PFS. Among patients with advanced solid tumors treated with ICIs, those who experienced immune side effects had higher DCRs and ORRs and demonstrated superior PFS. The observed benefit was more pronounced in patients with gastric, esophageal, and hepatocellular cancers.

**Key Points**

1. Patients who experience irAEs have better treatment outcomes after treatment with ICIs.
2. The observed benefit was more pronounced in patients with gastric, esophageal, and hepatocellular cancers.
3. No difference was observed between the benefits of patient efficacy and adverse immunological effects.

## 1. Introduction

In recent years, the incidence of malignant tumors has shown a year-on-year increase with the influence of various factors, such as environment and heredity, and death caused by tumors has become the second most common fatal disease.^[1]^ With an in-depth understanding of tumor pathogenesis and the development of drug discovery technologies, tumor immunotherapy has improved the treatment outcomes of many cancers, including melanoma and non-small-cell lung cancer. Nivolumab and Pembrolizumab, monoclonal antibodies targeting programmed cell death protein 1 (PD-1), have been approved for the treatment of advanced or recurrent unresectable gastroesophageal adenocarcinoma (GEA), esophageal squamous cell carcinoma (ESCC), lung cancer, and other cancers, and recent clinical trials have demonstrated that these drugs can prolong survival compared to cytotoxic chemotherapy, improve patient prognosis.^[2,3]^ Immune checkpoint inhibitors (ICIs) therapy is frequently associated with immune-related adverse events (irAEs), including thyroid dysfunction and dermatologic toxicities.^[4]^ Emerging evidence suggests a correlation between irAE development and improved survival outcomes following ICI treatment in malignancies such as melanoma and non-small cell lung cancer (NSCLC).^[5,6]^ These observations indicate that early-onset irAEs may serve as predictive biomarkers for enhanced therapeutic efficacy. Furthermore, appropriate management of irAEs may enable treatment continuity, thereby maximizing therapeutic exposure and potential clinical benefit.^[7]^

Prior research has established a correlation between irAEs and improved prognosis following ICIs therapy in NSCLC and hematologic malignancies. Emerging evidence extends this association to advanced renal cell carcinoma and gastric cancer, where irAE development correlates with superior clinical outcomes compared to irAE-negative patients.^[8,9]^ Nevertheless, comparative evidence regarding irAE prognostic significance across heterogeneous tumor types remains limited. No systematic investigation has yet determined whether this correlation represents a pan-tumor phenomenon or exhibits histology-specific variations.

This retrospective study therefore examines efficacy differentials and survival impact of irAE development in a multi-tumor cohort of advanced solid malignancy patients receiving ICIs. Our findings aim to inform clinical decision-making regarding ICI utilization and treatment response assessment.

## 2. Materials and methods

### 2.1. Case

This retrospective analysis included 59 patients with advanced solid tumors treated with ICIs at the Department of Medical Oncology, Anqing Petrochemical Hospital of Nanjing Drum Tower Hospital Group (August 2020–May 2022). The cohort comprised 44 males and 15 females, aged 40 to 81 years (mean 64.30 ± 9.96).

The inclusion criteria were as follows: patients with pathologically confirmed diagnosis of malignant solid tumors; patients with no pointer to radical surgical procedures or patients with local recurrence or distant metastasis after radical surgical procedures; patients who received more than 4 cycles of treatment with ICIs (or combination therapy with chemotherapy, antiangiogenic drugs, and targeted drugs); eastern Cooperative Oncology Group (ECOG) score of 0 to 2; Patients who gave informed consent and signed the informed consent form. The exclusion criteria were as follows: patients with autoimmune diseases; patients with abnormal immune function; incomplete clinical records, loss to follow-up, or voluntary withdrawal from the study protocol. All participants or legally authorized representatives provided documented informed consent. This study complied with the Declaration of Helsinki and received institutional review board approval from Anqing Petrochemical Hospital of Nanjing Drum Tower Hospital Group (Ethics Approval No. 202008) prior to commencement.

### 2.2. Treatment method

Treatment regimens comprised immune checkpoint inhibitor (ICI) monotherapy (n = 33) or ICI-chemotherapy combinations (n = 26). The ICI agents administered included: sintilimab, camrelizumab, tislelizumab, pembrolizumab, zimberelimab, penpulimab, and toripalimab. Concomitant cytotoxic agents consisted of docetaxel, nab-paclitaxel, carboplatin, gemcitabine, oxaliplatin, and tegafur. All ICIs were administered per manufacturer prescribing information at 3-week intervals, with dose modifications guided by treatment response and toxicity assessments.

### 2.3. Evaluation methodology

Tumor response was assessed via contrast-enhanced computed tomography per RECIST v1.1^[10,11]^ at baseline and every 8 weeks postcycle 1 initiation or upon symptomatic progression. Evaluations categorized responses as: complete response (CR), partial response (PR), stable disease (SD), or progressive disease (PD). Objective response rate (ORR) was defined as (CR + PR)/total patients × 100%, while disease control rate (DCR) comprised (CR + PR + SD)/total patients × 100%.

Treatment-emergent adverse events were documented throughout therapy and during the 28-day safety follow-up period using CTCAE v5.0 criteria. All participants underwent prospective monitoring for 12 months or until occurrence of death or radiologically confirmed disease progression.

### 2.4. Statistics

Statistical analyses were conducted using SPSS (v23.0; IBM corp., Armonk) and MedCalc (v20.100; MedCalc Software Ltd., Ostend, Belgium). Continuous variables with normal distribution were compared using independent *t*-tests, while non-normally distributed data were analyzed with Mann–Whitney *U* tests. Categorical variables were evaluated by χ^2^ or Fisher exact tests as appropriate. Progression-free survival (PFS) was analyzed via Kaplan–Meier methodology with log-rank testing, and multivariable Cox proportional hazards regression models were employed to identify independent prognostic factors. Survival curves were generated using MedCalc. A 2-sided α-level of 0.05 defined statistical significance.

## 3. Results

### 3.1. Baseline patient characteristics and distribution of patients with irAEs

Of all the 59 patients enrolled, there were 4 cases of gallbladder cancer, 15 cases of lung cancer, 4 cases of liver cancer, 9 cases of esophageal cancer, 11 cases of gastric cancer, 3 cases of colorectal cancer, 2 cases of breast cancer, 2 cases of squamous skin cancer, and 9 cases of other cancers (1 case of vertebral metastasis, 1 case of pancreatic cancer, 1 case of thymus cancer, 1 case of retroperitoneal lymph node metastasis, 1 case of renal cancer, 1 case of ovarian cancer, 1 case of malignant melanoma, 1 case of cervical cancer, 1 case of bladder cancer) There were 19 cases of first-line treatment, 21 cases of second-line treatment, 12 cases of third-line treatment, 4 cases of fourth-line treatment, and 3 cases of fifth-line treatment. Forty-six patients experienced irAEs and 13 patients did not, as shown in Table 1.

**Table 1:** Baseline characteristics of patients and distribution of patients with irAEs.

Variables		IrAEs (n = 46)	Non-irAEs (n = 13)	*x* ^2^ */t*	*P*
Sex (F/M)	[n (%)]	34/12	10/3	0.061	.804
Age		64.30 ± 9.96	71.69 ± 10.05	*t* = 2.356	.022
Tumor types (n)	Cholangiocarcinoma	2	2	4.749	.447
	Lung cancer	11	4		
	Esophageal cancer	6	3		
	Gastric cancer	9	2		
	Liver cancer	4	0		
	Others	14	2		
Drugs (n)	Sintilimab	11	2	9.545	.145
	Tislelizumab	14	4		
	Camrelizumab	14	3		
	Pembrolizumab	1	0		
	Zimberelimab	1	0		
	Penpulimab	1	1		
	Toripalimab	4	3		
Line (n)	First-line	15	4	0.355	.949
	Second-line	16	5		
	Third-line	9	3		
	≥Fourth-line	6	1		
Combination chemotherapy (n)	Yes	19	7	0.013	.979
	No	27	6		

### 3.2. Impact of the occurrence of irAEs on patient outcomes

Response evaluation revealed significantly superior outcomes in irAE-positive patients versus the non-irAE cohort. Among patients with irAEs (n = 46), we observed 14 PRs and 29 cases of SDs, yielding an ORR of 30.43% and DCR of 93.48%. Conversely, the non-irAE group (n = 13) demonstrated 1 PR and 4 SDs, corresponding to an ORR of 7.69% and DCR of 38.46%. Both ORR (χ^2^ = 3.647, *P* = .006) and DCR (χ^2^ = 5.664, *P* < .001) showed statistically significant enhancement in the irAE cohort, with detailed comparative data presented in Table 2. Further subgroup analyses showed that there was essentially no difference in the efficacy of patients treated with different ICIs (Tables S1 and S2, Supplemental Digital Content, https://links.lww.com/MD/P318), different treatment regimens (Tables S3 and S4, Supplemental Digital Content, https://links.lww.com/MD/P318), and different tumor types (Tables S5 and S6, Supplemental Digital Content, https://links.lww.com/MD/P318), further confirming that the occurrence of irAEs may be an important factor influencing patient outcomes.

**Table 2:** Therapeutic effect of irAEs patients after ICIs treatment.

Group	n	Therapeutic effect [n (%)]					
		CR	PR	SD	PD	DCR	ORR
IrAEs	46	0	14	29	3	93.48 (43/46)	30.43 (14/46)
Non-irAEs	13	0	1	4	8	38.46 (5/13)	7.69 (3/20)
		*Z* = 4.292				*x*^2^ = 5.664	*x*^2^ = 3.647
*P*		<.001				<.001	.006

### 3.3. Impact of the occurrence of irAEs on the PFS of patients

**Figure 1.**
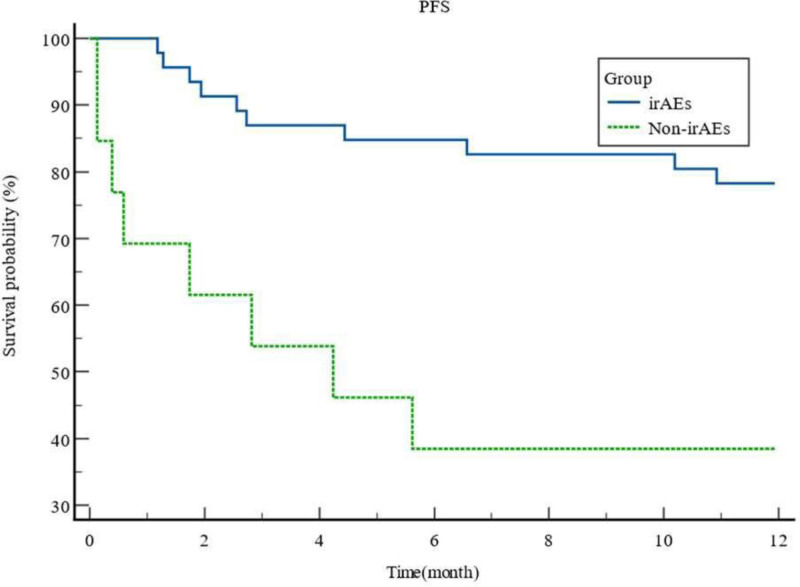
Survival analysis of PFS with and without the occurrence of irAEs.

According to Kaplan–Meier analysis (Fig. 1), follow-up was terminated at 12 months or upon occurrence of death/disease progression. The median PFS was significantly longer in patients developing immune-related adverse events (irAEs) compared to those without irAEs (10.34 months, 95% CI: 9.275–11.401 vs 5.82 months, 95% CI: 2.868–8.772; log-rank *P* = .0012).

In subgroup analyses focusing on irAE-positive populations, although the PFS remained comparable across various ICI types and therapeutic protocols (Figs. S1 and S2, Supplemental Digital Content, https://links.lww.com/MD/P318).

**Figure 2.**
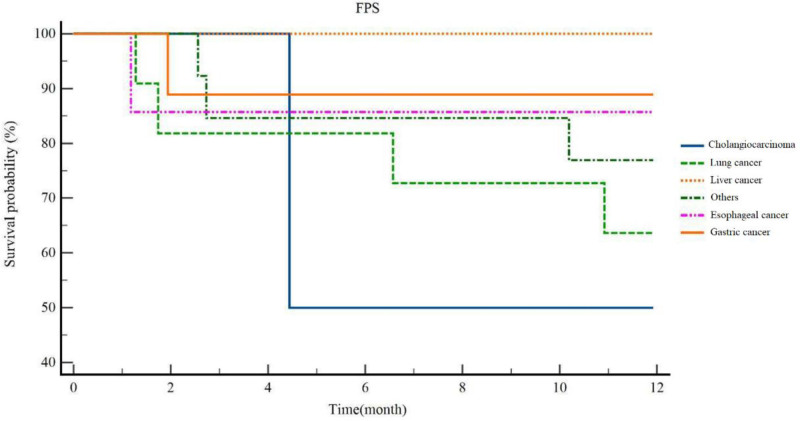
PFS survival analysis of patients with different tumor types in which irAEs occurred.

Kaplan–Meier analysis revealed significant heterogeneity in PFS among irAE-positive patients stratified by tumor type. The overall cohort (n = 46) demonstrated a median PFS of 8.22 months (95% CI: 2.981–13.459). Tumor-specific analyses showed the following median PFS durations: lung cancer: 9.50 months (95% CI: 6.867–12.135); esophageal carcinoma: 10.45 months (95% CI: 6.488–14.421); gastric cancer: 10.88 months (95% CI: 8.817–12.948); other malignancies: 10.42 months (95% CI: 8.318–12.525). Notably, no progression events occurred among hepatocellular carcinoma (HCC) patients during the 12-month follow-up period. Collectively, gastrointestinal malignancies (gastric cancer, HCC, and esophageal carcinoma) exhibited the most favorable PFS outcomes (Fig. 2).

### 3.4. Cox regression analysis of the effect of different irAEs on PFS in tumor patients

Multivariate cox regression analysis showed that any irAEs, cutaneous irAEs, and endocrine irAEs were significantly associated with increased PFS in patients, as shown in Table 3.

**Table 3:** Cox proportional hazards regression analysis of irAEs on PFS in tumor patients.

Survival	Univariable hazard ratio (95% CI)	*P*	Multivariable hazard ratio (95% CI)	*P*
Any irAEs	0.567 (0.325–0.960)	.04	0.542 (0.295–0.971)	.04
Skin irAEs	0.509 (0.264–0.916)	.02	0.476 (0.232–0.912)	.03
Endocrine irAEs	0.389 (0.064–1.248)	.13	0.237 (0.037–0.842)	.02

## 4. Discussion

The prognostic significance of immune-related adverse events (irAEs) during ICI therapy represents a critical consideration in immuno-oncology. While established associations between irAEs and survival benefits exist in melanoma, NSCLC, renal cell carcinoma, and gastric cancer,^[12–15]^ their prognostic relevance across diverse malignancies remains incompletely characterized. Furthermore, the efficacy and safety of ICI rechallenge following irAE development remain clinically uncertain,^[16]^ with potential variations in irAE profiles across antibody classes.^[17]^

This multi-tumor analysis demonstrates a significant association between irAE development and prolonged PFS in advanced solid tumors. IrAE-positive patients exhibited substantially superior median PFS (10.34 months; 95% CI: 9.275–11.401) versus irAE-negative counterparts (5.82 months; 95% CI: 2.868–8.772; log-rank *P* = .0012), corroborating prior tumor-specific observations. Notably: Enhanced disease control (DCR: 93.48% vs 38.46%; *P* < .001); Improved objective response (ORR: 30.43% vs 7.69%; *P* = .006). Previous studies have shown an association between irAEs and the outcome of immunotherapy in patients with melanoma, and 1 study showed that patients with NSCLC treated with immunotherapy had a longer duration of immune-related thyroid disease than patients without thyroiditis.^[18]^ Another study showed an association between skin irAEs and OS improvement in nivolumab-treated patients with NSCLC.^[19]^ This study showed an increase in PFS in the presence of endocrine or cutaneous irAEs using Cox multivariate analysis, which is consistent with previous findings.

Tumor-stratified analysis revealed differential survival benefits: Cholangiocarcinoma: 8.22 months (95% CI: 2.981–13.459); NSCLC: 9.50 months (95% CI: 6.867–12.135); esophageal carcinoma: 10.45 months (95% CI: 6.488–14.421); gastric cancer: 10.88 months (95% CI: 8.817–12.948). Gastrointestinal malignancies (gastric, hepatocellular, and esophageal carcinomas) demonstrated particularly enhanced clinical benefit from irAEs. These findings support irAE occurrence as a potential prognostic indicator across tumor types, correlating with superior treatment response. Gastrointestinal malignancies (gastric, hepatocellular, esophageal) exhibited the most pronounced clinical benefit, aligning with existing evidence of organ-specific immunobiological interactions.^[17]^

However, study limitations include restricted cohort size (n = 59) limiting subgroup analyses and restricting statistical power; heterogeneous tumor biology and treatment regimens; insufficient follow-up for overall survival assessment. Validation in larger prospective cohorts is warranted to establish irAEs as predictive biomarkers for ICI efficacy.

In this study, we observed that among patients who developed immune-related adverse events (irAEs), the therapeutic benefits were more pronounced in those with gastric cancer and hepatocellular carcinoma. The underlying reasons for this observation remain unclear, though it may be attributed to the inherent heterogeneity of the patient population. The relationship between irAEs and antitumor immune responses is complex and likely varies depending on several factors, including the specific organ affected by the irAE, the tumor histology, and individual patient characteristics. While some irAEs may reflect an immune response targeting antigens shared between tumor cells and normal tissues, other irAEs could be unrelated to the antitumor immune response altogether. Further investigation is required to better understand these intricate dynamics.

## 5. Conclusion

In conclusion, the development of irAEs has the potential to predict survival outcomes in patients with different tumors treated with ICIs. The development of irAEs should be carefully monitored after initiating ICI therapy to ensure longer patient survival.

However, this investigation has several methodological limitations that warrant consideration. The primary constraints stem from the relatively small sample size and incomplete dataset, which may compromise the statistical power of our analyses. Particularly, the restricted number of cases limited our capacity to conduct adequately powered subgroup analyses or perform more sophisticated multivariate assessments.

Despite these limitations, this preliminary study provides valuable exploratory insights into immunotherapy for patients with tumors. Our findings establish a foundational framework for future research directions, highlighting critical areas that require verification through large-scale population studies. Subsequent investigations should prioritize multicenter collaborations and prospective designs to enhance sample diversity, ensure comprehensive data collection, and enable robust stratified analyses across clinically relevant subgroups.

## Author contributions

**Conceptualization:** Cheng Zhao.

**Data curation:** Cheng Zhao, An Wang, Bin Zhai, Bin Jiang, Lina Hu, Hui Yu.

**Formal analysis:** Cheng Zhao, Cui Bai, Kai Chen.

**Funding acquisition:** Cheng Zhao.

**Investigation:** Cheng Zhao.

**Methodology:** Cheng Zhao.

**Project administration:** Cheng Zhao.

**Supervision:** Huiqian Liu.

**Writing – original draft:** Cheng Zhao.

## Supplementary Material


