# Supplementary material for: Prognostic differences in patients with advanced solid tumors receiving immune checkpoint inhibitors: The role of immune-related adverse events
Source: Medicine (Baltimore). 2025 Jul 4;104(27):e43153. doi: 10.1097/MD.0000000000043153 (PMC12237319; doi:10.1097/MD.0000000000043153)

**Prognostic Differences in Patients with Advanced Solid Tumors Receiving Immune Checkpoint Inhibitors: The Role of Immune-Related Adverse Events**

Cheng Zhao*, Huiqian Liu, An Wang, Bin Zhai, Bin Jiang, Lina Hu, Hui Yu, Cui Bai, Kai Chen

Department of Medical Oncology, Anqing Petrochemical-Hospital, Nanjing Drum Tower Hospital Group, Anging 246000, Anhui, China.

* Corresponding author: Cheng Zhao, 13866089591@163.com. Anqing Petrochemical Hospital of Nanjing Drum Tower Hospital Group.

**Contents:**

Comparison of efficacy of different ICIs and occurrence of irAEs vs non-irAEs.…………………………………………………………………………………………………S2

Comparison of efficacy of different tumor types and occurrence of irAEs vs non- irAEs…………………………………………………………………………………………………S3

Comparison of efficacy of different treatment programs and occurrence of irAEs vs non- irAEs.…………………………………………………………………………………………………S4

PFS survival curves of patients with different treatment regimens and different ICIs ………………S5

**Table S1 Comparison of efficacy of different ICIs**

| Group | Therapeutic effect [n(%)] | | | | | |
| --- | --- | --- | --- | --- | --- | --- |
|  | CR | PR | SD | PD | DCR | ORR |
| Sintilimab | 0 | 4 | 8 | 1 | 92.31(12/13) | 7.69(4/13) |
| Tislelizumab | 0 | 2 | 13 | 3 | 83.33(15/18) | 11.11(2/18) |
| Camrelizumab | 0 | 5 | 8 | 4 | 76.47(13/17) | 29.41(5/17) |
| Pembrolizumab | 0 | 1 | 0 | 0 | 100.00(1/1) | 100.00(1/1) |
| Zimberelimab | 0 | 1 | 0 | 0 | 100.00(1/1) | 100.00(1/1) |
| Penpulimab | 0 | 0 | 1 | 1 | 100.00(1/1) | 0 |
| Toripalimab | 0 | 2 | 3 | 2 | 71.43(5/7) | 28.57(2/7) |
| Total | 0 | 15 | 33 | 11 | 72.88(43/59) | 25.42(15/59) |

**Table S2 Differences in efficacy between patients who developed irAEs and those who did not develop irAEs among patients treated with different ICIs.**

| Group | irAEs | | | Non-irAEs() | | |
| --- | --- | --- | --- | --- | --- | --- |
|  | PR | SD | PD | PR | SD | PD |
| Sintilimab | 4 | 7 | 0 | 0 | 1 | 1 |
| Tislelizumab | 2 | 12 | 1 | 0 | 1 | 2 |
| Camrelizumab(n=17) | 4 | 8 | 1 | 1 | 0 | 3 |
| Pembrolizumab | 1 | 0 | 0 | 0 | 0 | 0 |
| Zimberelimab | 1 | 0 | 0 | 0 | 0 | 0 |
| Penpulimab | 0 | 0 | 0 | 0 | 1 | 0 |
| Toripalimab | 2 | 2 | 1 | 0 | 1 | 1 |
| Total | 14 | 29 | 3 | 1 | 4 | 8 |

**Table S3 Differences in efficacy among different tumor types of patients.**

| Group | Therapeutic effect [n(%)] | | | | | |
| --- | --- | --- | --- | --- | --- | --- |
|  | CR | PR | SD | PD | DCR | ORR |
| Cholangiocarcinoma | 0 | 2 | 1 | 1 | 75.0(3/4) | 50.0(2/4) |
| Lung Cancer | 0 | 2 | 11 | 2 | 86.67(13/15) | 13.33(2/15) |
| Esophageal Cancer | 0 | 3 | 4 | 2 | 77.78(7/9) | 33.33(3/9) |
| \|  \| [Gastric cancer](http://www.baidu.com/link?url=8bfOo8R7x7UGT8i5Ugrn6RF7mhsb0KhXjezK0EbfjC086VvPzixRTp2Gb0tMCXIYcsKx819yupo06z4LbjFk5LNIqvK1HmouiQKFTuqDJTuIhEqggPMzYE9VAvQkXCPY) \| \| --- \| --- \| | 0 | 4 | 5 | 2 | 81.82(9/11) | 36.36(4/11) |
| Liver cancer | 0 | 1 | 3 | 0 | 100.0(4/4) | 25.0(1/4) |
| Others | 0 | 3 | 9 | 4 | 75.0(12/16) | 18.75(3/16) |
| Total | 0 | 15 | 33 | 11 | 72.88(43/59) | 25.42(15/59) |

**Table S4 Differences in efficacy between patients who developed irAEs and those who did not develop irAEs among different tumor types of patients.**

| Group | irAEs |  |  | Non-irAEs | | |
| --- | --- | --- | --- | --- | --- | --- |
|  | PR | SD | PD | PR | SD | PD |
| Cholangiocarcinoma | 2 | 0 | 0 | 0 | 1 | 1 |
| Lung cancer | 1 | 10 | 0 | 1 | 1 | 2 |
| Liver cancer | 1 | 3 | 0 | 0 | 0 | 0 |
| Gastric cancer | 4 | 5 | 1 | 0 | 0 | 1 |
| Esophageal cancer | 3 | 3 | 1 | 0 | 1 | 1 |
| Others | 3 | 8 | 1 | 0 | 1 | 4 |
| Total | 14 | 29 | 3 | 1 | 4 | 8 |

**Table S5 Differences in efficacy among different treatment programs of patients.**

| Group | Therapeutic effect [n(%)] | | | | | |
| --- | --- | --- | --- | --- | --- | --- |
|  | CR | PR | SD | PD | DCR | ORR |
| ICIs monotherapy(n=33) | 0 | 8 | 17 | 7 | 75.75(25/33) | 24.24(8/33) |
| ICIs +chemotherapy(n=26) | 0 | 7 | 16 | 4 | 88.46(23/26) | 26.92(7/26) |
| Total(n=59) | 0 | 15 | 33 | 11 | 81.36(48/59) | 25.42(15/59) |

**Table S6 Differences in efficacy among different treatment programs of patients.**

| Group | irAEs | | | Non-irAEs | | |
| --- | --- | --- | --- | --- | --- | --- |
|  | PR | SD | PD | PR | SD | PD |
| ICIs monotherapy | 8 | 14 | 3 | 0 | 2 | 4 |
| ICIs +chemotherapy | 6 | 15 | 0 | 1 | 2 | 4 |
| Total | 14 | 29 | 3 | 1 | 4 | 8 |

**Figure S1 PFS survival curves of patients with different treatment regimens**

**
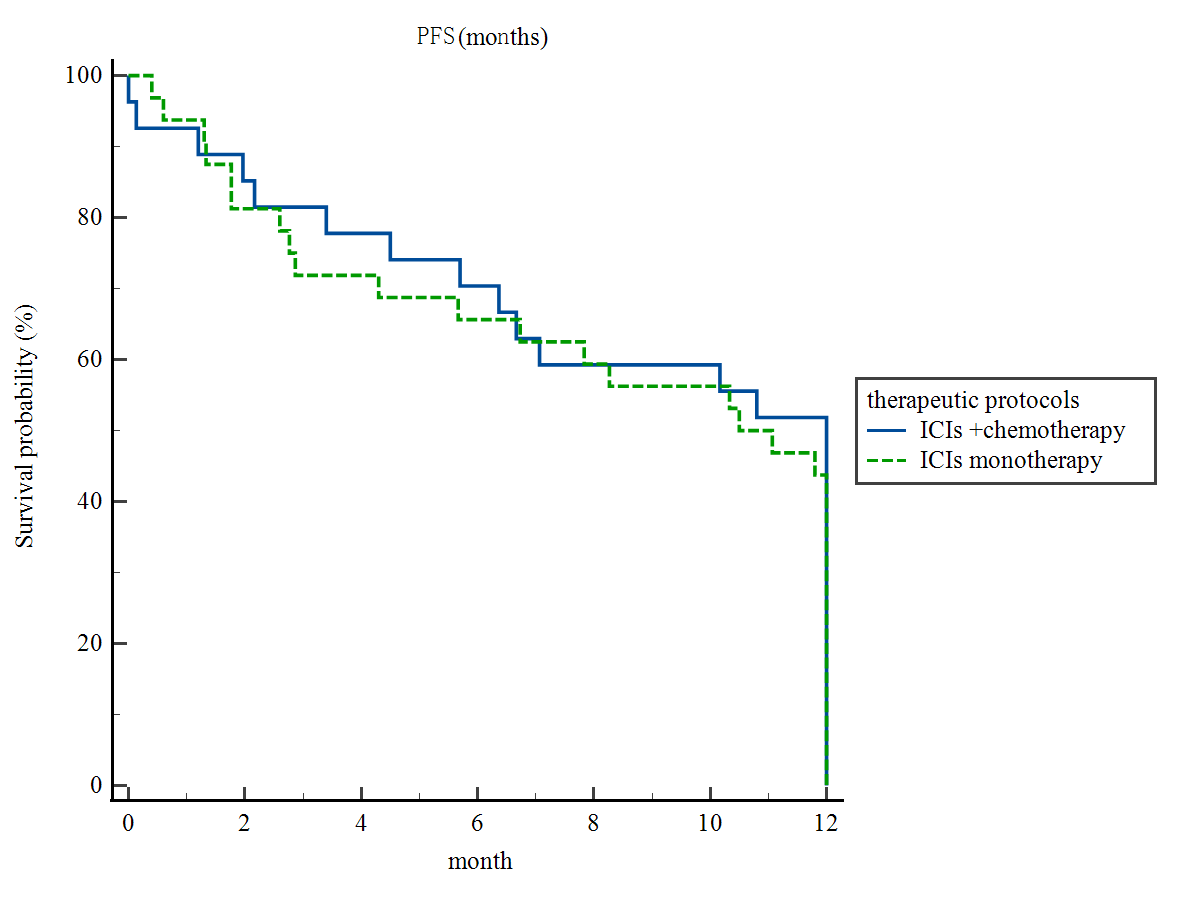
**

**Figure S2 PFS survival curves of patients with different ICIs**


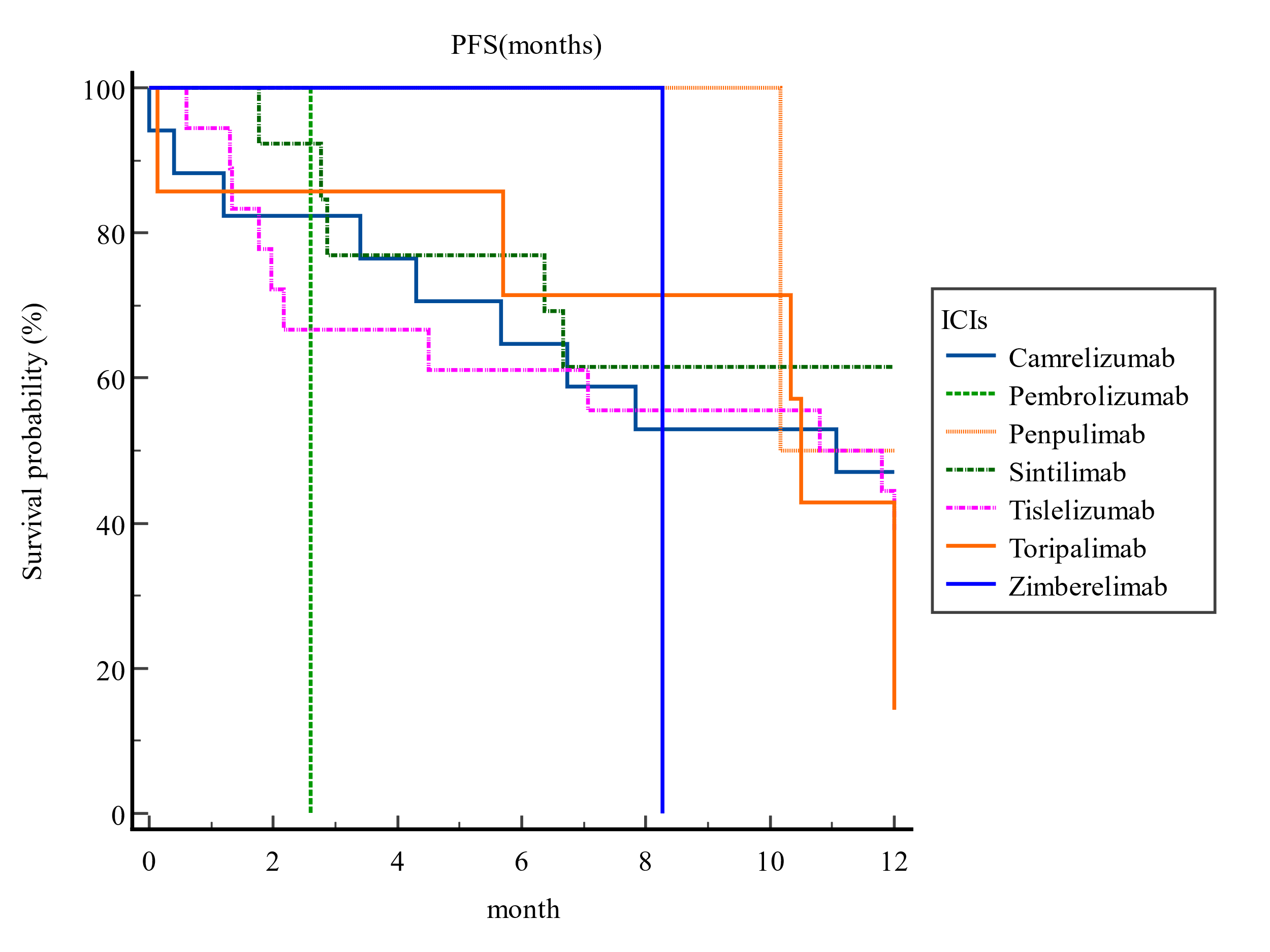

Supplement: Supplementary file 1 [file medi-104-e43153-s001.docx]
